# The development and validation of a Real Time Location System to reliably monitor everyday activities in natural contexts

**DOI:** 10.1371/journal.pone.0171610

**Published:** 2017-02-14

**Authors:** Gaby Judah, Jessie de Witt Huberts, Allan Drassal, Robert Aunger

**Affiliations:** 1 Environmental Health Group, London School of Hygiene and Tropical Medicine, London, United Kingdom; 2 Center for advanced Studies in Adaptive Systems (CASAS), Washington State University, Pullman, Washington, United States of America; Waseda University, JAPAN

## Abstract

**Introduction:**

The accurate measurement of behaviour is vitally important to many disciplines and practitioners of various kinds. While different methods have been used (such as observation, diaries, questionnaire), none are able to accurately monitor behaviour over the long term in the natural context of people’s own lives. The aim of this work was therefore to develop and test a reliable system for unobtrusively monitoring various behaviours of multiple individuals within the same household over a period of several months.

**Methods:**

A commercial Real Time Location System was adapted to meet these requirements and subsequently validated in three households by monitoring various bathroom behaviours.

**Results:**

The results indicate that the system is robust, can monitor behaviours over the long-term in different households and can reliably distinguish between individuals. Precision rates were high and consistent. Recall rates were less consistent across households and behaviours, although recall rates improved considerably with practice at set-up of the system. The achieved precision and recall rates were comparable to the rates observed in more controlled environments using more valid methods of ground truthing.

**Conclusion:**

These initial findings indicate that the system is a valuable, flexible and robust system for monitoring behaviour in its natural environment that would allow new research questions to be addressed.

## Introduction

The ability to measure and understand behaviour is vital for many disciplines, including psychology, behavioural science, public health and gerontology. This measurement and understanding is crucial for the design of effective interventions and assessment of their effectiveness. However, measuring behaviour accurately and without bias is difficult, particularly for many of the behaviors of interest in these fields: the assessment of hygiene behaviour, prosocial behavior, eating behaviour and environmental behaviour tends to be prone to social desirability bias, reactivity, and is often hampered by privacy concerns. As a consequence, the study of *actual behaviour* is often limited. In 2007, Baumeister and colleagues called for a renewed focus on behaviour in psychology, to prevent the discipline becoming just the science of “self-reports and finger movements” [1]. An additional limitation of relying on self-report and proxies of behaviour is that for many behaviours to achieve the desired health or other benefits, they need to be performed regularly and consistently, and thus require long-term assessment. Recent developments in sensor network technologies have resulted in a range of devices that could offer a solution for these limitations. Here, we describe the development and testing of a system to unobtrusively and reliably monitor behaviour over prolonged periods of time.

### Current methods for behaviour measurement

Self-report measures in the form of questionnaire- or interview-based responses in which people report on the frequency and circumstances of their own (past) behaviour are probably the most widely used tool to record behaviour. Although easy and cheap to administer, they have several crucial drawbacks. Firstly, people tend to vastly over-report desirable behaviour [2–6]. Secondly, people are unlikely to accurately remember the frequency or the details of their behaviour (e.g. [7–9]. Behaviours that are regularly performed and habitual—and thus not always available to introspection—are particularly prone to this kind of recall bias. [10–11].

A form of self-report measure that minimizes the likelihood of recall bias are diary measures where people report on their behaviour over a shorter time frame, usually daily. However, these are still vulnerable to socially desirable responding, and also burdensome to complete over the long term, thus risking substantial missing data. Furthermore, as self-monitoring has been shown to increase behaviour (e.g. [12–13]), the use of diary measures constitutes a behavioural intervention rather than just a method of measurement.

An alternative to self-report is to use observers to record people performing a behaviour, either in their natural environment or in a laboratory setting. However, behaviour monitored in public spaces or in a lab setting is unlikely to provide an accurate representation, as people tend to behave differently in public compared to when alone, and when in artificial lab situations rather than in the natural context for the behaviour. For example, the presence of others affects food intake (e.g. [14]) and handwashing with soap [15]. Moreover, if participants are aware that their behaviour is being observed, this introduces reactivity [16]. For certain behaviours, such as personal hygiene, visual observation could also violate privacy concerns. Also, as direct observation is expensive and intrusive [16, 17], it is not practical for the long-term monitoring of behaviour. Long-term monitoring is particularly important when investigating changes in behaviour or responses to an intervention, as it can take many months before a new behaviour becomes sustained (e.g., habitual) [18, 19].

To overcome the limitations of current behaviour measurement methods a system is needed that can: A) measure behaviour as unobtrusively as possible to minimize reactivity; B) work within private settings such as households as this where the behaviours of interest take place, and is likely to provide the most genuine reflection of a behaviour; and C) monitor behaviour over a prolonged period of time and is therefore sufficiently robust to stay in place without maintenance for several months. In addition to these requirements, a behaviour monitoring system ideally also should also be able to: D) identify different individuals, including in situations in which two people may be engaged in the same activity. The identification of individuals would not only enable associating behavioral results with psychological variables, it would also allow testing of the role of the social context as research has indicated that the adoption and performance of behaviour can depend on social influence, for example that tooth-brushing is a habit which is best inculcated in children by parents who serve as role models [20]. Finally, a behaviour measurement system should: E) be able to monitor several different activities as this would allow the determination of temporal sequences of activities. This is important as particular occasions sometimes matter much more to public health than others—for example, hands are much more likely to be contagious and require washing with soap after defecation than after other activities, and a recent study showed that flossing is more likely to be adopted and maintained if it is practiced after, rather than before, tooth-brushing [21]. Systems which only measure single behaviours cannot shed light on the behavioural context. All these criteria should be met in a system that is scalable and cost-efficient. These desired requirements of a behaviour monitoring system are summarised in Table 1.

**Table 1:** Desirable characteristics of a behaviour monitoring system.

Criteria	Requirements
Unobtrusive	Compact and relatively unobtrusive sensors
Works in a variety of everyday settings	Acceptable to target individuals Some degree of privacy
Can identify individuals	Able to identify individuals in situations likely to involve multiple people
Can measure multiple specific behaviours	Able to identify specific behaviours of these individuals Flexible enough for application to a variety of behaviours
Long-term measurement	Capable of being left for several months (e.g. long battery life and remote data monitoring) Reliable and robust (e.g. waterproof) Acceptable to householders
Pragmatic	Easy to install in multiple locationsStraightforward data output for ease of activity recognition Relatively low cost Relatively low technical training or skill-base to use
Reliable	Able to produce consistent measurements across periods and locations

Happily, technological progress could now make this holy grail of behaviour research possible. Developments in monitoring technologies have resulted in a range of devices that could be used by researchers to capture behavior. While different systems have been used for behavioural monitoring, none of these seem suitable for identifying specific, individual behaviour in the natural context of participants’ own homes, rather than in a model environment [22]. The aim of this work was therefore to develop and test a reliable system for unobtrusively monitoring and identifying specific behaviours of multiple individuals within the same household over a period of several months. Specifically, the target behaviours for this research were key hygiene behaviours that typically occur within the bathroom.

### Review of existing monitoring systems

Existing types of monitoring systems can broadly be categorized into rich data formats such as video and microphone and sensor systems, including non-wearable sensor systems and sensor systems that comprise solely or partially of wearable sensors. To identify the most suitable candidates for a reliable behaviour monitoring system, we assessed available solutions according to their match with the criteria in Table 1.

#### Video and microphone

**Video**. The most direct form of activity recognition system is video. However, this approach poses many problems. The data is very rich and therefore complex, and so is difficult to analyse automatically and therefore costly in terms of analysis and data storage. Possibly the biggest barrier to the use of a video system is privacy concerns. Though systems have been developed which preserve privacy to some extent, such as only recording silhouettes, many people would still be wary of having a video system installed in their homes [23].

**Microphones**. Microphones can also provide a rich source of data, as several activities have a specific sound signature. Stationary omnidirectional microphones have been used to measure activities in the bathroom [24]. However, due to the impact of environmental differences (e.g. room size, layout, materials of surfaces), the activity recognition algorithm would need to be trained in each setting, which would be impractical for a system intended to be scalable. Moreover, microphones could probably not differentiate between behaviour of two individuals in close proximity and will have quite poor recognition for common household activities such as using the washing machine, flushing the toilet and brushing teeth. Given these limitations, video and microphones do not meet our requirements.

**Non-wearable sensor systems**. Another class of monitoring system involve environmental sensors. The most straightforward of these are those which monitor a particular device or situation, such as medication adherence [3, 25], or monitoring of latrine use in India [26]. The greatest drawback of these is the lack of individual level data and that they cannot provide knowledge of the context or sequence of activities due to only one object being monitored.

Several classes of sensors track locations of people within a space. This can be done using motion sensors on the ceiling (e.g. [27, 28]), or pressure mats on/under the floor or items of furniture (e.g. [28]). Through learning patterns of typical behaviour, algorithms can learn to identify individuals (e.g. [27–29]), however this requires extensive training, and recognition can still be poor when there are several people in one setting [28]. In addition, location tracking systems cannot detect what activities are being performed even if a person is detected in a particular area. Motion sensors such as these have been combined with reed switches (a binary sensor measuring open or close states which can be easily fitted to drawers, doors etc.) to attempt to detect behaviours being performed. While this can provide fairly good recognition of higher level activities such as personal hygiene or cooking, it cannot determine more detailed information about object usage [30], so is not sufficient to monitor specific behaviours.

**Wearable sensor systems**. The most common type of wearable sensors monitor the activity level or posture of individuals. Several devices are incorporated into, or make use of, technology already present on mobile phones to identify whether participants are doing activities such as sitting, walking, running or climbing stairs (e.g. [31–33]). Other types give information on overall energy expenditure (e.g. [34, 35]). However, these applications to measure posture and energy expenditure cannot shed light on specific behaviours being carried out or the specific context in which activity takes place. Therefore these systems are not suitable for monitoring specific activities.

**Combination sensor systems**. From consideration of the systems above, we conclude that to achieve both identification of individuals and recognition of specific activities, it is necessary to combine wearable sensors with sensors in the environment. Common approaches following this model are Radio Frequency Identification (RFID) and Real Time Locating Systems (RTLS).

**RFID**. RFID uses electromagnetic fields to identify and track tags attached to objects [22, 36, 37]. This involves a wearable RFID reader (usually on the hand or wrist) and passive RFID tags on objects. While the RFID tags have the advantages of being small, cheap and easy to install, they tend to have a poor level of behaviour recognition [22]. The presence of metal or water can interfere with the signals, and the signal tends to be occluded by the human body [30, 38]. So for objects with a small grasping surface, where people are likely to hold them with their hand covering the RFID tag, the signal will often not be received which limits the application to fairly large objects. Furthermore, object uses are not detected if someone uses the hand not wearing the bracelet to perform them [22]. Therefore, RFID systems are currently not sufficiently reliable for our purposes.

**RTLS**. Another system that combines wearable sensors with environmental sensors are Real Time Location Systems (RTLS). RTLS use networked sensor technologies to locate people or assets, and track their movements in factories, hospitals and a variety of other commercial settings. An RTLS can log the physical location of an action, what happened before or after the target behaviour, identify the actor, and track such phenomena in multiple people simultaneously in real time, over the long term and without interference. RTLS would therefore meet all the requirements of the desired behaviour monitoring system

While there are several commercially available RTLS systems which can monitor people and objects, they still have certain drawbacks. Commercial systems are often very expensive, making them infeasible for typical research budgets, or have an inflexible system of location detection, which cannot easily be applied to different situations. For example, some sensors are disposable as they are designed for single use for patients in hospitals and only have a battery life of around two weeks. These are therefore not a cost-effective option for research, and unsuitable for longer term applications. There are systems which can detect motion of sensors, yet the method of location is not very sensitive (e.g., to room level), making it difficult to distinguish between people in the same approximate location. However, one commercially available system, the *Elpas II system*, is both affordable and well-placed to meet the requirements of being able to identify specific behaviours of particular individuals within confined spaces over the long term. (The system is commercially available from Elpas Solutions, part of Tyco International; see www.elpas.com).

### The selected system

The Elpas II system is used commercially to track the movement of staff and equipment in hospitals, provide security for items in museums, and track movement of goods in warehouses. The Elpas system comprises sensors (Healthcare Positioning Tags) which are worn on the wrist by individuals; the same sensors can be affixed onto objects. These sensors record whether or not they are in motion, and also whether they are in range of specified “zones”, or when they leave those zones (therefore the data is in binary form). These zones are created by LF (low frequency) Exciters, which are wired to a nearby plug socket. Zones can be set at four intervals between 0.15m and 1.5m in diameter. All motion and location data is received by a device called a Radio Frequency (RF) Reader.

This system was selected as it is relatively low cost, and fulfilled the essential criteria of being able to monitor behaviour of multiple individuals within a household, using relatively small sensors to be worn and affixed to objects. The system is flexible and can be tailored to different types of behaviour. As a commercial system, it is well tested and robust (e.g., the sensors are waterproof), and does not require frequent charging or replacing of batteries (estimated battery life of sensors is up to four years), thus allowing long periods of undisturbed monitoring without the need for maintenance visits. The data can also be transmitted wirelessly, allowing many households to be monitored simultaneously with minimal logistical difficulty, enabling any problems to be rapidly identified and resolved. While there is a significant one-time up-front capital cost (approximately £1200 per household for a full set of equipment), running costs are negligible and the system can be used for years in many households for a variety of studies (due to the system’s flexibility), such that the cost per behavioural observation becomes relatively low.

Adaptation of RTLS for use in naturalistic circumstances allows key behaviors to be monitored, thereby enabling intervention development and outcome measurement. Here, we describe the development and the testing of the systems’ ability to monitor individual behaviour in real households. This project is, to our knowledge, one of the few uses of sensor systems in real-life contexts for monitoring multiple behaviours in multiple individuals.

### Development of the research system

This study aims to demonstrate the validity of the system’s outputs through the monitoring of activities within the bathroom. Therefore, the existing system was adapted to be able to detect specific bathroom behaviours such as tooth brushing, soap use and toilet use—later extended to include flossing and vitamin taking as well.

To enable the detection of small motions in relevant objects, Elpas programmed their commercial sensors with a lower motion sensitivity threshold, resulting in three different levels of motion sensitivity. To ensure that motion records were not overwhelmed due to noise, Elpas also modified the sensors so that all motion data was sent as a burst of three identical messages.

**Fig 1:**
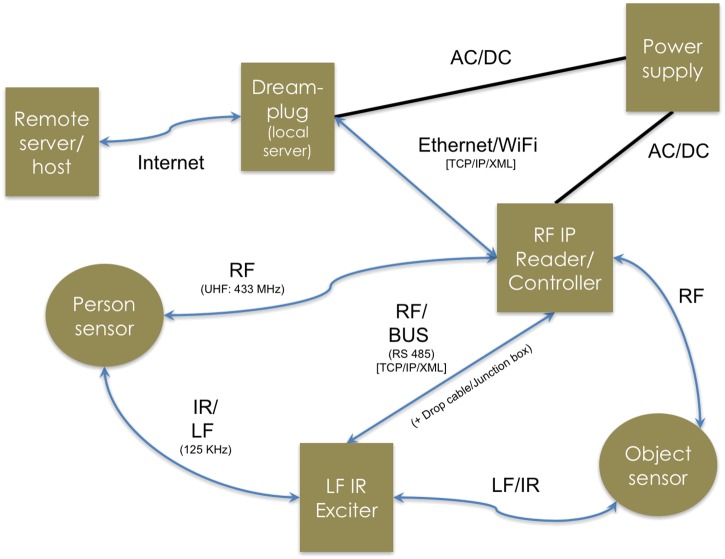
Elpas research system.

The sensors transmit data (motion start or motion stop, and zone information) wirelessly to a receiver called an RF (radio frequency) Reader. As this device does not have data storage capacity, the RF Reader was connected to a separate device—a Dreamplug computer (GlobalScale Technologies), programmed using Linux. The Dreamplug stored all data coming into the RF Reader, provided a time-stamp, compressed the data, and then uploaded it to a server on the university network via the internet. The university server account can be accessed via a password-protected website. This allowed remote, near real-time checking of the data and enabled any problems to be resolved promptly. To facilitate checking, the software was configured to send email alerts to the researcher if no data was being received from a particular site or sensor for a pre-defined time period. The programming of the Dreamplug, server database, and website was performed by staff from the CASAS group at Washington State University. System components and their communication protocols are shown in Fig 1.

#### Activity recognition

Although the Elpas commercial system includes software to interpret the sensor data in various ways, it was not fit for the activity recognition needs of this application. Therefore, an independent activity recognition program was designed as part of system development. This program was specifically written for this application in Python by CASAS personnel.

The basic strategy of the activity recognition algorithm is based on the assumption that actions can be determined by co-location of a person and moving object. For example, if a person’s tag is detected in the sink zone near the time that the toothpaste, tap and toothbrush cup moved, then it can be inferred that the person has brushed their teeth. In order to recognise behaviour from the sensor data, an activity recognition program was created that made use of a variant of the common ‘sliding window’-based logic [39]. It conducts the steps outlined below:

- Creates motion:start/motion:stop event pairings, along with a length of time that the sensor was in motion (i.e., the time between start and stop times).
- If there are any missing motion:start or motion:stop events (likely due to noise in the environment preventing some signals from getting through), then these are inferred using the value entered for the “-m” option (see Table 2). Missing motion:stop events are added either at “m” seconds following the motion:start, or one second prior to the next motion:start event, whichever interval is the shorter. This is in order to prevent the creation of overlapping motion:start/motion:stop pairs. Missing motion:start events are inferred in the same way, using an “m” second interval from the corresponding motion:stop event, or one second following the prior motion:stop event, according to whichever gives the shorter motion duration interval.
- “Window extensions” are then created by subtracting/adding the window extension times to the motion:start and motion:stop times respectively. If an object is classed as a “moveable object” (those for which use involves a change in place, e.g. toothpaste, floss), then this is using the number of seconds specified in the “g” option. If an object is classed as a “non-moveable object” (those for which use involves only a small vibration, e.g., toothbrush cup, toilet roll holder), then this is created using the “j” option.
- “Event windows” are then created by combining all events with overlapping “window extensions” into a single event window. In this way, all events are assigned to one of a number of event windows.
- The events contained within an event window are compared with the pre-defined activity sets (see Table 3). Points are awarded according to how well the combination of events in the window match with every activity listed.
- If the points assigned within a window for a particular activity exceed a predefined threshold, then that activity is deemed to have occurred.
- The data from the person-tags includes information about whether they were detected in an exciter zone. Therefore, if a person is detected in the zone where an activity is taking place during the duration of an event window, the activity is attributed to that person.

**Table 2:** Parameter values in activity recognition programme.

Parameter	Parameter label	Value (seconds)
Missing motion:start or motion:stop	m	2
Window extension for non-moveable objects	g	30
Window extension for moveable objects	j	15

**Table 3:** Parameter values for each behaviour within the activity set.

Behaviour	Threshold	Zone & Object	Motion point value
**Toothbrushing**	80	Bathroom sink zone	30
		Toothbrush cup	20
		Toothpaste	50
**Soap use**	80	Bathroom sink zone	34
		Soap dispenser	66
**Toilet use**	80	Toilet zone	30
		Toilet flush	50
		Toilet roll	20
**Flossing (HH3 only)**	75	Bathroom sink zone	25
		Toilet zone	25
		Floss/Floss picks	50
**Vitamin taking (HH3 only)**	75	Bathroom sink zone	25
		Bathroom toilet zone	25
		Tap	10
		Cup	10
		Vitamins	50

The parameters within the activity recognition process are specified in Table 2. These values were based on observation of performance or acting out of the key behaviours by the research team, and examination of the raw sensor data output. These parameters could be modified if desired, enabling the system to be flexibly applied to different environments and different behaviours.

The parameter values for each behaviour within the activity set are shown in Table 3. Each event window is compared with every behavior in the activity set, and points are awarded based on the correspondence between the events within the window and each of the behaviours. This allows determination of the events that occur within a window. The parameters within each behaviour in the activity set were specified so that detection of the minimum number of necessary events corresponding to a behaviour would result in detection of that behavior. To test the validity of the developed method, the system was subsequently tested in three households.

## Methods—system validation

### Sample

The system was set up in a convenience sample of three households. Inclusion criteria were that each household had to contain more than one individual, in order to test the capability of the system to distinguish between individuals (each household monitored had two people living there, both of whom took part in the study, thus in total the behavior of six people was monitored). The individuals being monitored also had to use just one bathroom, as the system was only set up in one bathroom per household. The study was explained to participants, any questions were answered, and they signed informed consent to participate in the study. Participants did not receive any remuneration for their participation. The study received ethical approval from the London School of Hygiene and Tropical Medicine.

### Sensor setup

**Fig 2:**
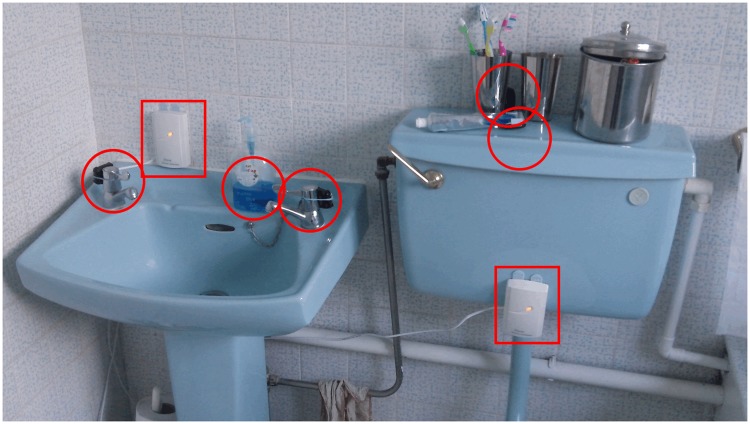
Elpas aystem in action.

The households were set up with the monitoring equipment by the researcher. The RF reader and Dreamplug were placed in an appropriate location outside the bathroom. This was selected to be close to plug sockets, relatively close to the bathroom. One exciter was placed as centrally as possible behind the sink (to mark the sink zone). The other exciter was affixed to the toilet cistern (to mark the toilet zone). The exciters were connected by a thin wire to a plug socket outside the bathroom. (See Fig 2 for a sample household setup; red circles indicate the placement of object sensors; rectangles indicate exciters.)

Sensors from the three different sensitivity levels were selected to reflect the different expected motion patterns of the different objects. In order to avoid false positives, each object was affixed with the least sensitive sensor which reliably detects actual object usage. For example, toothbrush cup use would only effect a slight movement, toilet flush a slightly greater movement, and toothpaste an even greater movement. By attaching sensors to these objects, the following behaviours were measured: going to the toilet, washing hands with soap, brushing teeth, flossing, and vitamin taking. As the optimal settings for the system were still being determined for the first two households to be set up, adjustments were made to the sensors following short periods of monitoring and visual comparison between the raw sensor data and the ground truth, until a higher level of accuracy was reached. Adjustments included substituting sensors with different levels of sensitivity, or changing the location of the exciter.

### Participant instructions

Participants were told to wear the sensors on their dominant wrist continuously during the trial. They were also given a behaviour recording sheet, which was left inside the bathroom. Every time they left the bathroom, participants were asked to fill in their ID number (self-selected to ensure anonymity), the date and time, whether anyone else was present in the bathroom at the same time, and which activities they undertook while in the bathroom: toilet, handwashing with soap, toothbrushing, flossing, vitamin taking and ‘other’ (e.g., showering). Following final adjustments, the participants in the three households recorded these behaviours for 43 days, 11 days and 19 days respectively. This record of behaviour was treated as the ‘ground truth’, and manually compared to the output from the activity recognition program (although of course it is expected that errors and omissions occurred with respect to the users recording their own behaviour).

### Analysis

In order to compare the activity recognition program’s performance to the ground truth, two independent raters reviewed the ground truth records to the output files from the activity recognition programme, and classified recognition for each of the monitored behaviours as being true positive, false positive or false negative. Precision was calculated as true positive/(true positive+false positive) and recall was calculated as true positive/(true positive+false negative). Precision gives an indication of the likelihood that a behaviour recognized by the program is a true activity as recorded in the ground truth, and recall gives an indication of the likelihood of behaviour from the ground truth being recognized by the program. The precision and recall rates were also split according to rater, household and behaviour.

To assess the validation method of comparing the ground truth with the data from the activity recognition program, the reliability of the results from the two raters for each of the behaviours were compared using the intra class coefficient (ICC; reflected on a scale of 0–1, with 1 indicating perfect inter-rater reliability). The mean precision and recall rates for the two raters was used to assess the ability of the sensor system and activity recognition program to correctly identify behaviour.

## Results

### Behaviours monitored

Toothbrushing, soap use and toilet use was monitored in all three households. Household 3 also monitored flossing and taking vitamin tablets.

### Reliability check

A two-way mixed consistency, average-measures intra class coefficient (ICC) was used to assess inter-rater reliability [40]. As can be seen in Table 4, the resulting ICCs were in the good to excellent range, ranging from .84 to 1, indicating that coders had a high degree of agreement in their comparisons of the ground truth with the output of the activity recognition programme.

**Table 4:** Intra-class coefficients for the reliability of the measurements for the different types of behaviour between the two raters.

	True positives	False Positives	False negatives	Precision	Recall
**Toilet Use**	.997	.891	.999	.871	.961
**Soap Use**	.891	.891	.999	.871	.961
**Flossing**	.947	.963	.998	.957	.993
**Totals**	.998	.912	.998	.841	.996

### Recognition rates

Table 5 shows the mean precision and recall rates across each household and behaviour. (The recognition rates, split by household, rater and behaviour, are shown in the S1 Table). Mean household rates were also calculated based on the three behaviours that were measured in every household (toothbrushing, soap use, toilet use). Precision rates were high, with scores over 70% across all behaviours and households, and above 95% for all common behaviours in household 3, above 80% for toothbrushing and toilet use in household 2, and for toilet use in household 1. The high precision rates indicate that most of the instances of the behaviours recognised by the system were correctly detected.

**Table 5:** Mean precision and recall rates across the two raters, split by behaviour and household.

	Household 1		Household 2		Household 3		Mean (per behaviour)	
**Behaviour**	Precision	Recall	Precision	Recall	Precision	Recall	Precision	Recall
**Toothbrushing**	78.8%	36.0%	85.7%	29.7%	96.9%	82.7%	87.13%	49.46%
**Soap use**	77.1%	24.1%	72.0%	79.0%	95.2%	95.2%	81.43%	66.1%
**Toilet use**	83.5%	82.3%	83.3%	18.9%	95.2%	60.8%	87.33%	54%
**Flossing**					51.5%	55.0%		
**Vitamin**					100.0%	93.3%		
**Mean (from common behaviours)**	79.8%	47.5%	80.3%	42.5%	95.8%	79.6%		

However, recall rates were lower, with values above 70% only seen in toilet use in household 1, soap use in household 2, and toothbrusing, soap use and vitamin taking in household 3. The remaining recall rates in households 1 and 2 were below 40%. This indicates that for many behaviours, the system possibly didn’t detect half of the behaviours that occurred in the bathroom. A possible explanation for the overall higher rates in household 3 was that this household was set up last, and so the system may have been installed in a more robust way

## Discussion

In this paper we have described the development and testing of a sensor system to monitor behaviour of individuals in their own households. The results of this trial in three households indicate that the system is robust and can monitor behaviour over a prolonged period of time in various circumstances, that it can identify various types of behaviour, and that it can attribute these behaviours to specific individuals within a household.

The observed precision rates were high, robust across behaviours, and—to a lesser extent—across households. Recall rates were comparatively low in two out of the three households and less consistent between the different behaviours. The large variation in recall levels across households as well as behaviours suggests that the recall capacity of the system is likely to be a consequence of the set-up of the system or the household environment, rather than generally sensitivity to a particular behaviour. That recall rates were very good in the last household, after additional researcher practice with the set-up of the system, suggests that with the correct set-up, good recall rates can also be achieved with this system.

Both the precision and recall rates achieved in this study are comparable to other similar systems. In one study of a short-range RFID-based system deployed in a model studio apartment in which subjects performed a randomized but known sequence of behaviours, precision values for 14 different everyday behaviours ranged from 72–100% while recall ranged from 40–100% [38]. Similarly, in a richly networked simulated studio apartment, in which three subjects performed a morning routine, precision ranged from 85–90% while recall ranged from 42–81% [41]. In another study, one participant performed a sequence of 15 activities four times, in the participant’s home or in a supermarket (for a shopping scenario), using video recording for ground truthing, while wearing a number of sensors. Precision rates in this case ranged from 26–97%, recall from 22–90% [42]. The closest parallel to the current study, however, investigated bathroom-related behaviours like brushing teeth, toileting and showering using a variety of sensor-types, with written diaries for ground-truthing, in two single-person households [43]. Precision and recall rates between 50–85% were achieved in that study, but it did not require the identification of individuals. Similar results were achieved by other studies [44,45]. It should be noted that these others systems were typically tested in model environments, often involved a single person conducting a specified routine, and that the ‘ground truth’ was often established through observation or video recording, rather than the participants written recall records (i.e., using a more reliable form of ‘ground truth’). The fact that comparable precision and recall rates were observed under ‘messy’ real-life circumstances, monitoring the behaviour of multiple persons over a long-term period (rather than a single demonstration), suggest that the current system is a viable method to enable the long-term monitoring of behaviour of individuals in their natural environment in a relatively unobtrusive way.

From a practical point of view, the system has also been shown to be robust, relatively cheap, and is relatively easy to install; it can thus be used by non-experts to collect data on relatively large samples of individuals. Moreover, the system is flexible and can be adapted to measure different behaviours. For example, the system has also been adapted to monitor behaviours such as hair washing, showering, shaving, cooking, household cleaning and doing the laundry (Aunger, pers. comm). Indeed, while the measurement periods, the number of monitored behaviours, the number of household members monitored and the overall sample size were small in this study, a subsequent study used this system to monitor multiple behaviours in multiple rooms in 56 households for a period of four months each, indicating that the system is flexible, practical and scalable [46].

To our knowledge, this is one of the few systems to date that can identify multiple micro-scale behaviours of individuals in a multi-person context without pre-identification of behaviour types. Because of its ability to identify individuals, to assess multiple types of behaviour, and its capacity for long-term behavioural monitoring, the system allows new kinds of research questions to be addressed, such as the relationship between psychological variables and behavioural outcomes, the nature of long-term behavioural processes such as habit formation, the influence of temporal and physical context, or the role of social influence on behaviours performed.

As this was the first attempt to develop such a system, there is a need for improvement as well. As mentioned, the recall rates were relatively low and should be improved before the system can be used to reliably measure behaviour. A necessary first step for this is to uncover what causes these low recall rates. The findings seem to suggest that aspects of the set-up of the sensor system in different homes is critical; more insight into what aspects of the setup are crucial will hopefully lead to more robust recall rates. Also, while the system is unobtrusive compared to other behaviour monitoring systems, some participants remained aware of being monitored such that reactivity to the system itself continues for longer than a week or so. In some cases, the sensors attached to the household objects are difficult to hide and a relatively invisible surface, and thus can serve as reminders to enact the desired behaviours. However, as the system can monitor behaviour over prolonged periods of time, is likely that people get used to the sensors attached to the objects and they no longer serve as a reminder after an initial adjustment period (e.g., [22]).

## Conclusion

To answer research questions in many disciplines, as well as for end-user applications such as life-logging or to assist with monitoring the daily living of vulnerable populations (such as in nursing homes), measuring behaviour unobtrusively under natural circumstances for considerable periods is highly desirable, but often beyond the capabilities of existing data collection systems. In one of the first ‘real-world’ tests of a system developed to measure multiple behaviours over relatively long periods of time in situations where multiple individuals are interacting, the Elpas II system proved reliable. It worked to identify the activities of individuals as well as more expensive systems tested under more controlled circumstances. This system thus allows the confident testing of scientific hypotheses in natural situations, allowing new kinds of research questions to be addressed, such as the effects of long-term behavioural processes (e.g. habit formation), the influence of temporal and physical context, the role of social influence, or the process of age-related decline, and do so without significantly influencing the process itself. It also permits the accurate measurement of the consequences of behavioural interventions, and should therefore find wide use in both the behavioural and implementation sciences.

## Supporting information

S1 Dataset Data household 1. (XLSX)

S2 Dataset Data household 2. (XLSX)

S3 Dataset Data household 3. (XLSX)

S1 Table Recognition rates for each household. (DOCX)
