# Supplementary material for: The development and validation of a Real Time Location System to reliably monitor everyday activities in natural contexts
Source: PLoS One. 2017 Feb 14;12(2):e0171610. doi: 10.1371/journal.pone.0171610 (PMC5308850; doi:10.1371/journal.pone.0171610)
Supplement: S1 Table — (DOCX) [file pone.0171610.s004.docx]

# **S1 Table**

**S1 Table: Recognition rates for each household, split by rater and behaviour.**

a. Household 1

| **Behaviour** | **Rater** | **TP** | **FP** | **FN** | **Total** | **Precision** | **Recall** |
| --- | --- | --- | --- | --- | --- | --- | --- |
|  |  |  |  |  |  |  |  |
| **Toothbrushing** | **1** | 21 | 5 | 39 | 65 | 80.8% | 35.0% |
|  | **2** | 20 | 6 | 34 | 60 | 76.9% | 37.0% |
| **Soap use** | **1** | 14 | 4 | 42 | 60 | 77.8% | 25.0% |
|  | **2** | 13 | 4 | 43 | 60 | 76.5% | 23.2% |
| **Toilet use** | **1** | 89 | 13 | 17 | 119 | 87.3% | 84.0% |
|  | **2** | 83 | 21 | 20 | 124 | 79.8% | 80.6% |

TP=True Positive, FP=False Positive, and FP=False Negative.

b. Household 2

| **Behaviour** | **Rater** | **TP** | **FP** | **FN** | **Total** | **Precision** | **Recall** |
| --- | --- | --- | --- | --- | --- | --- | --- |
|  |  |  |  |  |  |  |  |
| **Toothbrushing** | **1** | 6 | 1 | 17 | 24 | 85.7% | 26.1% |
|  | **2** | 6 | 1 | 12 | 19 | 85.7% | 33.3% |
| **Soap use** | **1** | 17 | 8 | 3 | 28 | 68.0% | 85.0% |
|  | **2** | 19 | 6 | 7 | 32 | 76.0% | 73.1% |
| **Toilet use** | **1** | 5 | 1 | 22 | 28 | 83.3% | 18.5% |
|  | **2** | 5 | 1 | 21 | 27 | 83.3% | 19.2% |

TP=True Positive, FP=False Positive, and FP=False Negative.

c. Household 3

| **Behaviour** | **Rater** | **TP** | **FP** | **FN** | **Total** | **Precision** | **Recall** |
| --- | --- | --- | --- | --- | --- | --- | --- |
|  |  |  |  |  |  |  |  |
| **Toothbrushing** | **1** | 31 | 1 | 7 | 39 | 96.9% | 81.6% |
|  | **2** | 31 | 1 | 6 | 38 | 96.9% | 83.8% |
| **Soap use** | **1** | 20 | 1 | 1 | 22 | 95.2% | 95.2% |
|  | **2** | 20 | 1 | 1 | 22 | 95.2% | 95.2% |
| **Toilet use** | **1** | 41 | 1 | 24 | 66 | 97.6% | 63.1% |
|  | **2** | 38 | 3 | 27 | 68 | 92.7% | 58.5% |
| **Flossing** | **1** | 9 | 8 | 6 | 23 | 52.9% | 60.0% |
|  | **2** | 8 | 8 | 8 | 24 | 50.0% | 50.0% |
| **Vitamin taking** | **1** | 14 | 0 | 1 | 15 | 100.0% | 93.3% |
|  | **2** | 14 | 0 | 1 | 15 | 100.0% | 93.3% |

TP=True Positive, FP=False Positive, and FP=False Negative.
